# Deep Learning Anomaly Classification Using Multi-Attention Residual Blocks for Industrial Control Systems

**DOI:** 10.3390/s22239084

**Published:** 2022-11-23

**Authors:** Jehn-Ruey Jiang, Yan-Ting Lin

**Affiliations:** Department of Computer Science and Information Engineering, National Central University, Taoyuan City 320317, Taiwan

**Keywords:** anomaly detection, anomaly classification, industrial control system, deep learning, deep neural network, multi-attention block, residual block

## Abstract

This paper proposes a novel method monitoring network packets to classify anomalies in industrial control systems (ICSs). The proposed method combines different mechanisms. It is flow-based as it obtains new features through aggregating packets of the same flow. It then builds a deep neural network (DNN) with multi-attention blocks for spotting core features, and with residual blocks for avoiding the gradient vanishing problem. The DNN is trained with the Ranger (RAdam + Lookahead) optimizer to prevent the training from being stuck in local minima, and with the focal loss to address the data imbalance problem. The Electra Modbus dataset is used to evaluate the performance impacts of different mechanisms on the proposed method. The proposed method is compared with related methods in terms of the precision, recall, and F1-score to show its superiority.

## 1. Introduction

The industrial control system (ICS) integrates information technology (IT) and operational technology (OT) to monitor, control, and manage network-interconnected devices in large-scale industrial production systems or critical infrastructures, such as manufacturing factories, power plants, waterworks, oil refineries, gas pipelines, and public transportation systems [1]. Once cyber attackers intrude into an ICS to launch attacks, its performance degrades and some functions may fail, leading to huge losses. For example, a Taiwan chipmaker was attacked by WannaCrypt malware in 2018. Consequently, many chip-fabrication factories were shut down, leading to a loss of about USD 256 million [2]. For another example, an American oil pipeline system was halted by a ransomware cyberattack, and consequently, a ransom of USD 4.4 million was paid to restore its operations [3].

Anomalies occur before or during major attacks are launched. It is therefore helpful to develop methods to detect and classify anomalies associated with cyberattacks. Alerts are issued once anomalies are detected and/or classified. Traditional anomaly detection and classification methods cannot be directly applied to ICS applications due to differences in protocols and attack types between traditional networks and ICS networks. Several studies proposed ICS anomaly detection and classification methods that inspect network packets of the Modbus and S7Comm protocols. Gomez et al. [4] proposed supervised and unsupervised machine learning methods to detect ICS anomalies. Ning et al. [5] proposed an anomaly detection method based on the generative adversarial network (GAN) model [6] and the deep neural network (DNN) model.

Jiang and Chen [1] proposed an ICS anomaly detection method (abbreviated as the JC-AD method in this paper) and an ICS anomaly classification method (abbreviated as the JC-AC method in this paper). The two methods first utilize the denoising autoencoder (DAE) [7] to reduce data noise and extract core features from packets. Then, the JC-AD method employs the synthetic minority oversampling technique (SMOTE) [8] and the Tomek link (T-Link) [9] mechanism to oversample and undersample data for dealing with imbalance packets, where the majority of class samples (i.e., normal packets) significantly outnumber the minority class samples (i.e., anomalous packets). The SMOTE and the T-LINK mechanisms are for the binary-class samples, so they are not employed by the JC-AC method that addresses multi-class samples. Finally, both methods use extreme gradient boosting (XGBoost) [10] based on the ensemble learning concept to avoid overfitting to achieve good performance.

Among all the above-mentioned ICS anomaly detection methods, the JC-AD method [1] has perfect (i.e., 100%) accuracy, precision, recall, and F1-score. The JC-AC method [1] is the sole ICS anomaly classification method; it has almost perfect (i.e., nearly 100%) precision, recall, and F1-score.

This paper proposes an ICS anomaly classification method integrating difference mechanisms to improve the performance of the JC-AC method. The proposed method is flow-based; that is, it investigates the flow of packets instead of a single packet for classifying anomalies. It builds a DNN with multi-attention blocks [11] for spotting core features, and with residual blocks [12] for avoiding the gradient vanishing problem. The DNN is trained with the Ranger [13] (i.e., RAdam [14] + Lookahead [15]) as the optimizer to prevent the training from being stuck in local minima, and with the focal loss [16] to address the data imbalance problem. The proposed method can be used in conjunction with the JC-AD method. Specifically, it can be used for better anomaly classification after the JC-AD method perfectly detects ICS anomalies. Moreover, the proposed method can also be used for detecting ICS anomalies when viewed as a binary-class (i.e., normal-anomalous) classifier. As will be shown, it has comparably good performance in detecting ICS anomalies.

The Electra Modbus dataset [17] reported in [4] is employed to evaluate the proposed method’s performance. As the proposed method integrates several mechanisms, the evaluation also assesses the performance impact of not using a single mechanism. Furthermore, the evaluation results are compared with those of the JC-AC method to show that the proposed method indeed improves the JC-AC method in terms of the precision, recall, and F1-score of anomaly classification. Notably, the proposed method is shown to have comparably good ICS anomaly detection performance when compared with related methods.

The contribution of this paper is three-fold. First, it proposes a novel flow-based method integrating the mechanisms of the muti-attention block, residual block, Ranger optimizer, and focal loss to construct a DNN for monitoring ICS network packets to classify anomalies. Second, extensive experiments using the Electra Modbus dataset are conducted to evaluate the performance impacts of different mechanisms. Third, the performance of the proposed method is compared with those of related methods to show its superiority.

The rest of this paper is organized as follows. Section 2 describes background knowledge. Section 3 elaborates the proposed method. Performance evaluation and comparisons of the proposed method, with related methods, are shown in Section 4. Finally, Section 5 concludes the paper.

## 2. Background

### 2.1. Multi-Attention Block

**Figure 1:**
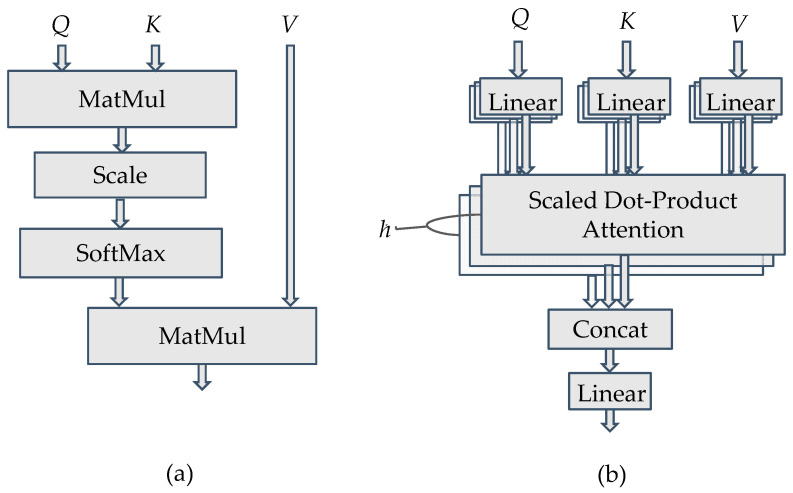
(**a**) The scaled dot-product attention block, and (**b**) the multi-attention block.

The attention mechanism [11] utilizes the attention block to assign different weights to each part of the DNN input, and extract more critical information for achieving better performance. It is widely used in applications such as machine translation, voice recognition, and image recognition, etc. The structure of the attention block (or the scaled dot-product attention block) is shown in Figure 1a. Based on the input vector, the attention mechanism obtains query vector *Q* through the Query matrix, key vector *K* through the Key matrix, and value vector *V* through the Value matrix. The attention score can be obtained by multiplying *Q* and *K*, then scaling and normalizing the product by the SoftMax function to obtain the attention weight, which is in turn multiplied by *V* to produce the output. Unlike recurrence-based models, such as the recurrent neural network (RNN), which have to sequentially check each input vector one by one, the attention mechanism can check all input vectors simultaneously to determine which input vector has a higher attention score to be paid more attention to. It thus has better performance than its counterparts. If the attention mechanism considers many queries, it is called a multi-attention mechanism and can be realized by multi-attention blocks, as shown in Figure 1b. It can be regarded as running through the attention mechanism multiple times (say, *h* times) in parallel. Each running of the attention mechanism can pay attention to different parts of input vectors to have an independent output result. All the attention output results are subsequently concatenated and linearly transformed to be the final output. 

### 2.2. Residual Block

**Figure 2:**
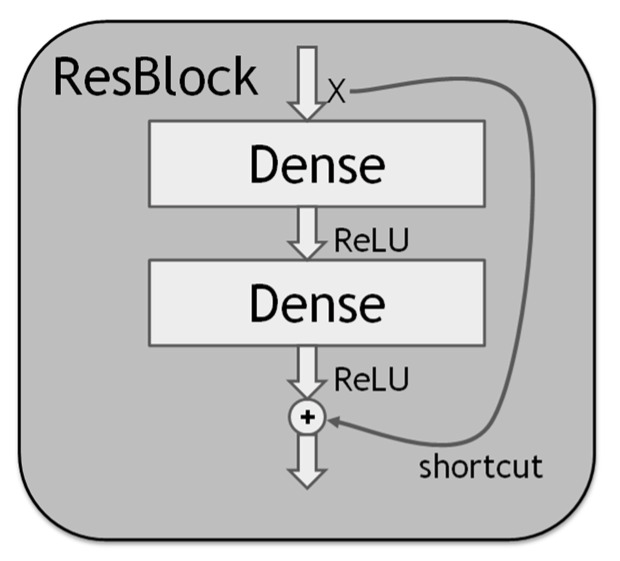
The residual block structure.

Increasing the depth of a DNN usually improves its performance. However, the depth increase causes the gradient vanishing problem so that the gradient approaches zero; it also causes the gradient exploding problem so that the gradient becomes excessive. The two problems may make the DNN weight update insensitive to output changes; thus, it is sometimes difficult for the DNN training error to converge. Although batch normalization can mitigate the two problems, there still exists the degradation problem that deeper DNNs may have worse performance than shallower DNNs. The residual block (ResBlock) [12], whose structure is shown in Figure 2, can be used to alleviate all the problems, as described below. The ResBlock has the normal dense layer with the ReLU (Rectified Linear Unit) or another activation function. In particular, it has the shortcut connection of identity mapping. With the shortcut, the ResBlock can learn the residual (i.e., difference) between the input and the output of the block. It can thus focus on the residual, which is more significant. As such, it is more sensitive to output changes and can mitigate the gradient vanishing, and the gradient exploding problems, which in turn can alleviate the degradation problem.

### 2.3. RAdam

RAdam [14] stands for Rectified Adam; it is simply an Adam optimizer [18] with a warmup scheme. Note that Adam stands for the Adaptive Moment Estimation, a well-known gradient descent optimization scheme in the DNN error-backpropagation process. On the one hand, in the early stage of training a DNN with the Adam optimizer, the variance of training errors of all samples is relatively small. However, after several epochs, the variance of training errors of all samples grows large. The reasons are as follows. If the warmup scheme is not employed and a large learning rate is used in the early stage of training a DNN model, the model becomes overfitting to the few samples ever seen. Thus, the training error is quite large for unseen samples. On the other hand, the Adam optimizer with the warmup scheme can reduce the variance of training errors of all data samples, as the learning rate is small in early stages and then grows in later stages of training the DNN. This can prevent the training from getting stuck in local minima for getting better performance.

**Figure 3:**
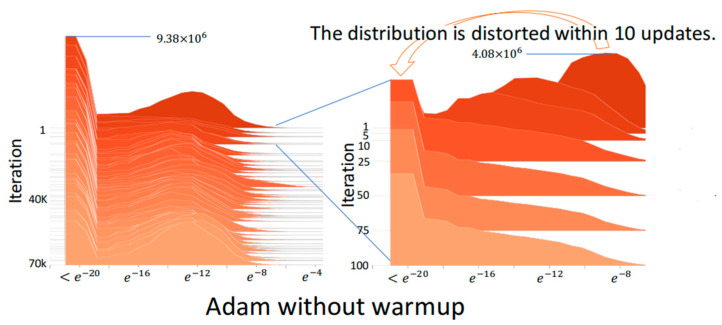
The absolute gradient histogram during training a DNN using Adam without warmup for machine translation on the IWSLT2014 De-En dataset [14].

**Figure 4:**
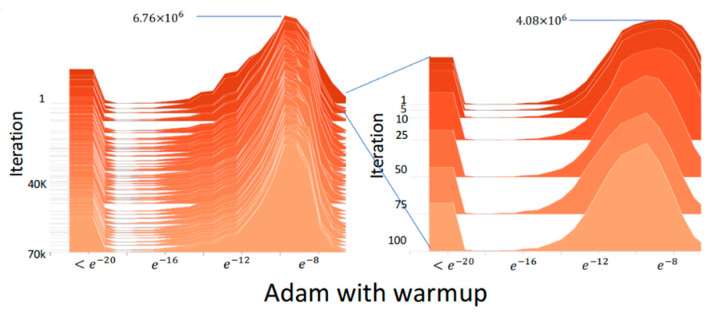
The absolute gradient histogram during training a DNN using RAdam (i.e., Adam with warmup) for machine translation on the IWSLT2014 De-En dataset [14].

Figure 3 shows the absolute gradient histogram during training a DNN using Adam without warmup for machine translation on the IWSLT2014 German-English (De-En) dataset [14]. In the histogram, the *y*-axis is the iteration (epoch), and the *x*-axis is the absolute gradient value in a logarithmic scale, whose height stands for the frequency. It can be observed from the histogram that using Adam without warmup makes the gradient distribution distorted to have mass centers of relatively small values in the first 10 iterations, which indicates the training may fast converge and be trapped in local minima after the first few iterations. Figure 4 shows the absolute gradient histogram during training a DNN using RAdam (i.e., Adam with warmup) for machine translation on the IWSLT2014 De-En dataset [14]. It can be observed from the histogram that the gradient distribution is not distorted after the first few iterations. This can avoid the bad situation that the training fast converges and is trapped in local minima.

### 2.4. Lookahead

The core concept of Lookahead [15] is to prepare two sets of weights for the neural network, one set of fast weights and one set of slow weights. When the fast weights are updated *k* times, the slow weights are updated to half the extent of the fast weights. In this way, even if the fast weights get stuck in local minima, the slow weights can still escape local minima to achieve better performance. Note that the Lookahead mechanism and the above-mentioned RAdam mechanism are combined as the Ranger optimizer [13] for training DNNs for improving performance.

**Figure 5:**
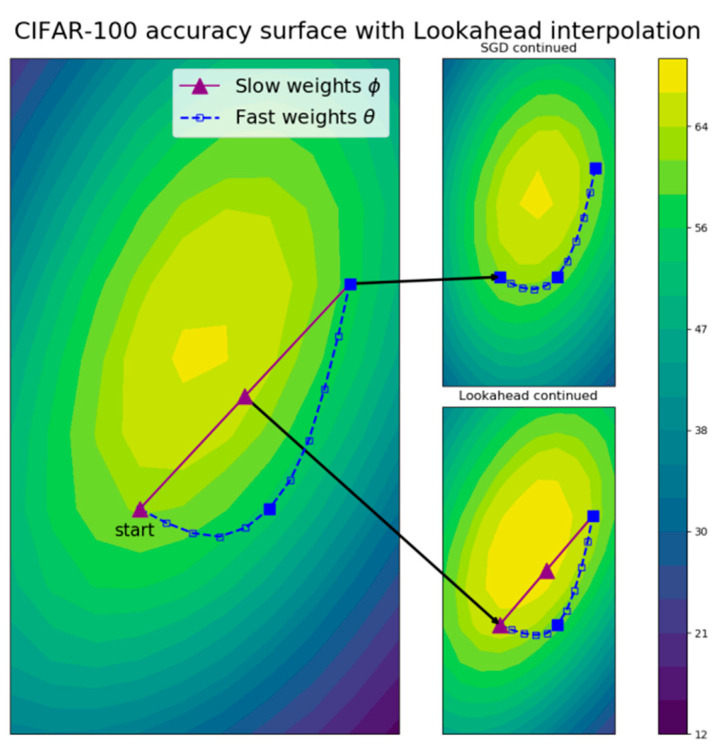
The visualization of Lookahead effects with *k* = 10 through a ResNet-32 test accuracy surface at epoch 100 on the CIFAR-100 dataset [15].

Figure 5 [15] is the visualization of Lookahead effects with *k* = 10 for training the ResNet-32 model [12] using stochastic gradient descent (SGD) as an image classifier on the Canadian Institute For Advanced Research-100 (CIFAR-100) dataset [19]. The figure shows the test accuracy surface of the model using fast weights indicated by the blue-dashed path, and slow weights indicated by the purple-line path. It can be observed from Figure 5 that Lookahead can quickly progress to the global minima than SGD.

### 2.5. Focal Loss

The core concept of the focal loss [16] is to set the loss of correctly classified samples (i.e., simple samples) to be small, and to set the loss of misclassified samples (i.e., difficult samples) to be large. Equation (1) is for the simplified cross entropy (CE), where *p_t_* is the probability to correctly predict the input sample to be positive. Equation (2) is for the focal loss (FL), where *α_t_* is the parameter related to data imbalance, and *γ* is the parameter related to the difficulty of sample detection and classification.

(1) $$\mathrm{CE}\left(p_{t}\right)=-\log p_{t}\;$$

(2) $$\mathrm{FL}\left(p_{t}\right)=-\alpha_{t}{\left(1-p_{t}\right)}^{\gamma }\log p_{t}\;$$

**Figure 6:**
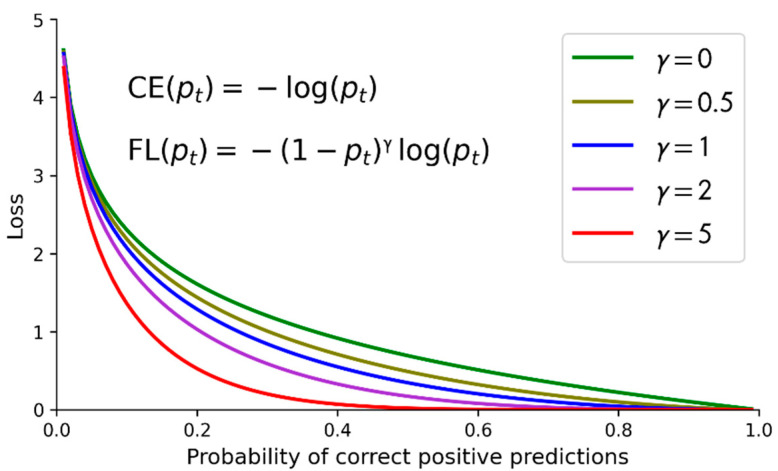
The comparisons of the CE and the FL with different values of *γ*.

Figure 6 shows the CE and the FL with data imbalance parameter α*_t_* = 1 (for not considering data imbalance) and *γ* = 0, 0.5, 1, 2, and 5 [16]. Note that the CE is actually the FL with *γ* = 0. The *y*-axis is the loss, and the *x*-axis is *p_t_*, the probability to correctly predict the input sample to be positive. It can be observed from Figure 6 that the well-classified samples or examples (i.e., those with *p_t_* larger than 0.5 and even close to 1) are associated with the FL that fast approaches 0 when *p_t_* grows. The FL approaches 0 faster than the CE. It can also be observed that the FL sets the loss of well-classified examples to be small, and sets the loss of misclassified examples to be large.

## 3. Proposed Method

**Figure 7:**
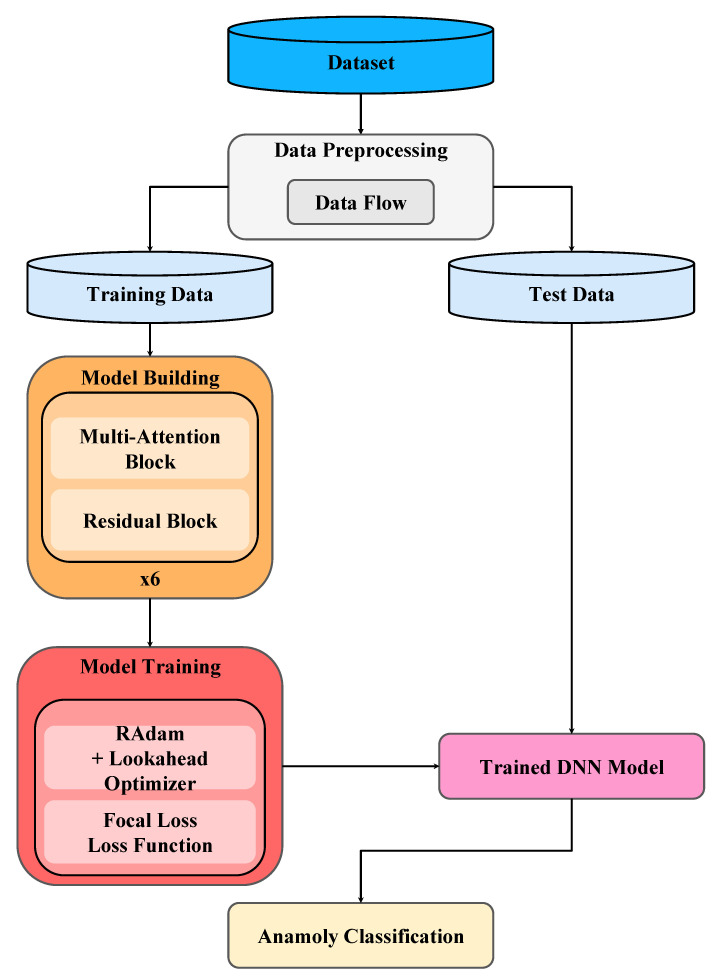
The workflow of the proposed method.

The workflow of the proposed method mainly consists of three steps: data preprocessing, model building, and model training, as shown in Figure 7. Each step is elaborated in a subsection below.

### 3.1. Data Preprocessing

The data preprocessing contains four sub-steps: label encoding, normalization, data flow processing, and data splitting. The four sub-steps are described below.

1. Label encoding:

In this sub-step, non-numeric features (e.g., categorial features) are converted into integers. If the number of categories of a feature is *N*, then the feature is converted into an integer in the range of [0, *N* − 1]. Using integers to represent non-numeric features may cause the problem that the DNN model mistakes the order relations among integers as the precedence relations among different categories, which in turn may influence the model performance. The one-hot encoding can avoid the problem by inserting *N* new columns and by assigning 1 in one column, and 0 in the other columns for a category. However, one-hot encoding needs many extra columns, whereas label encoding does not. Since there are likely many categories in the ICS anomaly classification setting, label encoding is adopted to convert non-numerical features.

2. Normalization:

Numerical features may have different ranges, which influence the training of neural networks. The proposed method adopts the min-max normalization to scale values of a feature into the range of [0, 1], while the distribution of feature values remains the same. With normalization, features are transformed to be within a common scale without distorting their distributions, which in turn can improve DNN model training stability and model performance.

**Figure 8:**
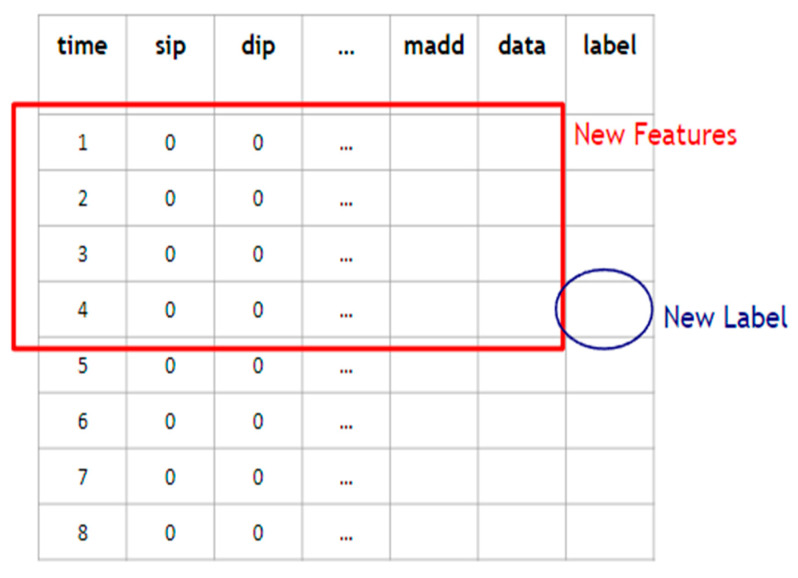
New features and a label are derived from a sliding window of *s* = 4 consecutive packets of a flow.

3. Data flow processing:

If two packets have an identical pair of the source IP address and the destination IP address, then they are considered to belong to the same flow. Packets of the same flow are sorted by timestamps. As shown in Figure 8, new features are derived form a sliding window of *s* (say, *s* = 4) consecutive packets of the same flow, and the label of the *s*th packet is taken as the new label associated with the new features. In this way, the new flow-based data are derived. When an attacker launches an attack, he/she usually sends multiple malicious data packets to the same target device, causing anomalies. Therefore, finding related packets with the same source and destination IP addresses through the data flow processing is very helpful for anomaly classification.

4. Data splitting:

New flow-based features and associated labels of data are split into the training dataset, the validation dataset, and the test dataset according to the ratios of 0.6, 0.2, and 0.2. The datasets are used to train, validate, and test data. Specifically, the training dataset is used to train a DNN model to have a small error, and the validation dataset is applied to the trained model to check (or validate) if the error is still small to prevent the model from overfitting to the training dataset. The above-mentioned actions constitute an iteration or an epoch of the training process. The training process stops when the error keeps decreasing and stays very small or when the maximum number of iterations is reached. Afterwards, the best model with the smallest error ever encountered is output as the final model. At last, the test dataset is applied to the final DNN model for assessing the model performance.

### 3.2. Model Building

**Figure 9:**
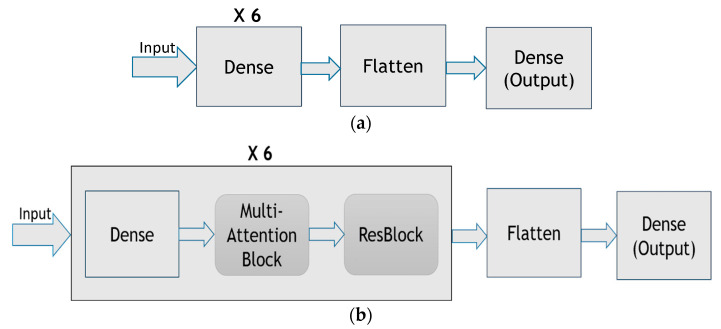
(**a**) The DNN without multi-attention blocks or residual blocks, (**b**) the DNN with multi-attention blocks and residual blocks adopted by the proposed method.

The proposed method builds the DNN that combines multiple attention blocks and residual blocks. Figure 9a shows the DNN without multi-attention blocks or residual blocks, whereas Figure 9b shows the DNN with multi-attention blocks and residual blocks adopted by the proposed method. In the DNN shown in Figure 9b, the first layer is the input layer. Then, there are *t* (say *t* = 6) copies of the dense layer, the multi-attention block layer, and the residual block layer, with each layer having eight neurons. The flatten layer and the dense layer then follow. The last is the output layer. In total, the DNN has 51 layers, with the last layer using the SoftMax, and the other layers using Swish [20] as the activation function. It is shown that the Swish function can help mitigate the gradient vanishing problem [20]. The He normal initializer [21] is employed to initialize neural weights. The initializer draws samples from a truncated normal distribution and is shown to have good performance.

### 3.3. Model Training

The parameters for training the DNN model are described as follows. The focal loss is used as the loss function with the parameter *α* set as 0.25, and the parameter *γ* set as 2, which are suggested by [16]. Ranger is used as the optimizer, and the minimum and the maximum learning rates are set as 0.000001 and 0.001, respectively. The early stopping scheme with the parameter patience set as 32 is employed to early stop the model training when the training loss and the validation loss no longer decrease. This can prevent the DNN model from overfitting.

## 4. Performance Evaluation

The Electra Modbus dataset [17] is applied for evaluating the performance of the proposed method in terms of some metrics. This section first introduces the dataset, describes the metrics, demonstrates the performance evaluation results, and then shows performance comparisons of the proposed method and related methods.

### 4.1. The Electra Modbus Dataset

Electra Modbus dataset [17] reported in [4] collects Modbus TCP protocol packet data generated during normal and abnormal operation of an electric traction substation used in the railway industry. It uses an ICS testbed to gather data. The testbed is composed of a supervisory control and data acquisition (SCADA) system, a firewall, a switch, a programmable logic controller (PLC) master, four PLC slaves, some ICS devices, and a special device that can launch man-in-the-middle (MitM) attacks for generating anomalous network packets. The special device launches attacks, records the features of all packets, and labels packets as normal or anomalous for 12 hours for generating the dataset. Recorded features of the Electra Modbus dataset are the timestamp, source MAC address, destination MAC address, source IP address, destination IP address, request, function code, error, memory address, and data sent between the PLC master and slaves, as shown in Table 1.

Packets generated during the period of normal operations are labelled as normal, whereas packets generated during the period of attacks are regarded as anomalous. There are in total 5.2% of anomalous packets associated with three categories of attacks, namely, reconnaissance, false data injection, and replay attacks. As shown in Table 2, the three categories of attacks are further classified into seven classes, as described below. The “function code recognition attack” is launched by generating malicious packets to scan all possible function codes of the attacked PLC. Attackers inject fake packets for performing the “read attack” or “write attack” on the PLC. The “response modification attack” and the “force error in response attack” are launched by modifying the response of a slave device. The “command modification attack” is launched by modifying command packets of a master device. The “replay attack” is launched by retransmitting packets ever sent by the master or slave devices. The ratios of various attack classes are also shown in Table 2.

### 4.2. Performance Metrics

The performance of the proposed method is evaluated in the following metrics: the precision, recall, and F1-score, as defined in the following equations. Note that in the equations, TP (True Positive) stands for the number of classifying anomalous packets as anomalous ones; TN (True Negative) stands for the number of classifying normal packets as normal ones; FP (False Positive) stands for the number of classifying normal packets as anomalous one, and FN (False Negative) stands for the number of classifying anomalous packets as normal ones.

This paper evaluates the performance of the proposed method in terms of only the precision, recall, and F1-score. This is because many existing ICS anomaly classification methods, such as those proposed in [1], also adopt the three metrics to evaluate their performance. In order to compare with related methods properly, the proposed method also adopts the three metrics for its performance evaluation.

(3) $$\mathrm{Precision}=\frac{\mathrm{TP}}{\mathrm{TP}+\mathrm{FP}}$$

(4) $$\mathrm{Recall}=\frac{\mathrm{TP}}{\mathrm{TP}+\mathrm{FN}}$$

(5) $$F1-\mathrm{score}=2\times \frac{\mathrm{Precision}\times \mathrm{Recall}}{\mathrm{Precision}+\mathrm{Recall}}$$

### 4.3. Performance Evaluation and Comparison

**Figure 10:**
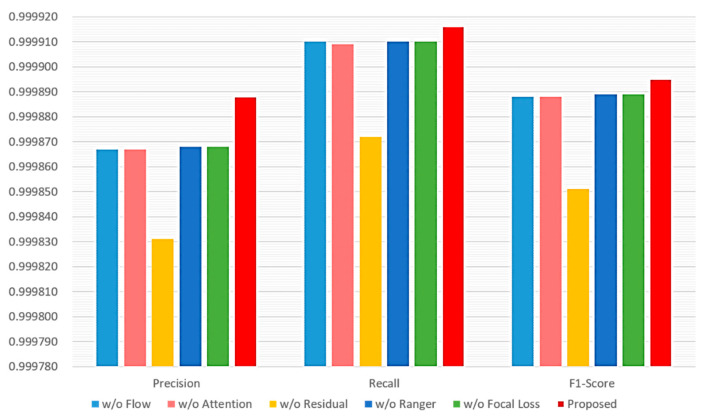
Performance impact of various schemes adopted by the proposed method.

The proposed method utilizes many mechanisms. First, it uses the flow-based data investigation mechanism. It also uses the DNN with the mechanisms of the multi-attention block, the residual block, the Ranger optimizer, and the focal loss. The performance impact of not adopting a single mechanism is assessed. Figure 10 shows the performance evaluation of six different cases of mechanism combinations. In case 1, flow-based data investigation mechanism is not used; instead, per-packet investigation mechanism is used. Case 2 omits the muti attention block mechanism, whereas case 3 omits the residual block mechanism. Case 4 uses the Adam optimizer to replace the Ranger optimizer. The cross-entropy, instead of the focal loss, is used in case 5. All mechanisms are used in case 6. It can be observed that the combination of all mechanisms has the best performance. Furthermore, the residual block mechanism has the most impact on performance, as not using it leads to the worst performance. As to other mechanisms, they have less and similar impacts on performance than the residual block mechanism.

**Figure 11:**
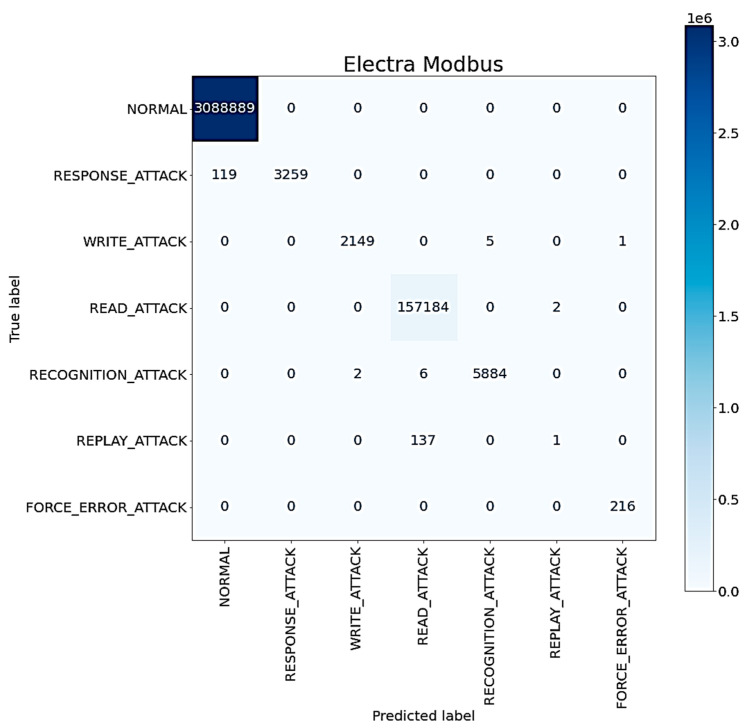
The confusion matrix of the proposed ICS anomaly classification method.

The confusion matrix of the proposed ICS anomaly classification method using all mechanisms is shown in Figure 11. It can be observed that most anomalies can be classified correctly. However, some anomalies, especially those associated with the replay attack and the read attack, are misclassified. This is probably due to the fact that most replay attacks behave similarly to read attacks; that is to say, most replay attacks read data illegally [4].

**Figure 12:**
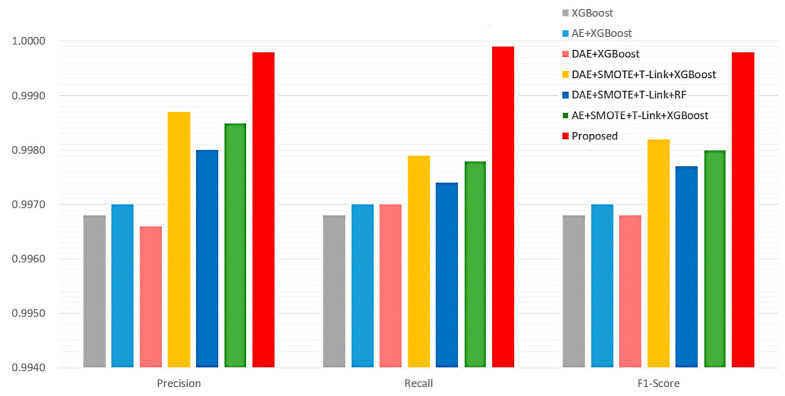
Performance comparisons of the proposed method and related ICS anomaly classification methods for the multi-class case.

The performance evaluation results of the proposed method are compared with those of the related ICS anomaly classification methods for the multi-class case, as shown in Figure 12 and Table 3. Based on Figure 12 and Table 3, it can be observed that the proposed method is better than the JC-AC method in terms of the precision, recall, and F1-score.

As mentioned earlier, the proposed method can also be used for detecting ICS anomalies when viewed as a binary-class (i.e., normal-anomalous) classifier. For the binary class case, the precision, recall, and F1-score of the proposed method are all 0.9999, which is quite high. Table 4 shows the comparison results of the proposed method with related ICS anomaly detection methods for the binary-class case. The methods for comparison are the JC-AD method [1] and the methods based on the support vector machine (SVM) [4], the one class support vector machine (OCSVM) [4], the random forest (RF) [4], the isolation forest (IF) [4], the DNN [4], and the integration of GAN and DNN (GAN + DNN) [5]. The proposed method is inferior to the JC-AD method, which has the perfect performance; however, it almost outperforms all other related methods in all performance metrics like the precision, recall, and F1-score.

## 5. Conclusions

This paper proposes a flow-based ICS anomaly classification method. The method first obtains new features based on the flow of packets. It then employs multi-attention blocks for spotting core features, and uses residual blocks for alleviating the gradient vanishing problem. Furthermore, it adopts the Ranger optimizer to avoid the overfitting problem and to accelerate the convergence of the DNN training. The focal loss is finally used as the loss function to deal with the data imbalance problem.

The Electra Modbus dataset is used to evaluate the performance of the proposed method. It is observed that the residual block has the most impact on the performance of the proposed method. The proposed method is shown to outperform the JC-AC method in terms of the precision, recall, and F1-score for ICS anomaly classification. When viewed as a binary-class (i.e., normal-anomalous) classifier, the proposed method can also be used for detecting ICS anomalies. For the binary class case, the proposed method also has comparably high performance of 0.9999 in the precision, recall, and F1-score metrics.

In the future, we plan to apply the proposed method to other ICS environments, such as those under different types of attacks, such as distributed denial-of-service (DDoS), and those using various protocols such as S7 Communication (S7Comm), and Distributed Network Protocol 3 (DPN3), etc. Furthermore, we also plan to employ new techniques, such as graph neural networks, to improve the ICS anomaly detection and classification performance.

## Figures and Tables

**Table 1:** Descriptions of the Electra Modbus dataset [4].

Feature	Description	Data Type
time	Timestamp	String
smac	Source MAC address	String
dmac	Destination MAC address	String
sip	Source IP address	String
dip	Destination IP address	String
request	Indicates whether the packet is a request (packet from master to slave)	Boolean
fc	Function code	Integer
error	Indicates whether there has been an error in reading/writing operation	Boolean
madd	Memory address to perform read/write operation	Integer
data	In the case of a read operation, it indicates the data that the slave sends to the master. In the case of a write operation, it indicates the data that the master sends back to the slave	Integer
label	Label for attacks and normal samples	String

**Table 2:** Attack classes of the Electra Modbus dataset [4].

Classes	Percentage of Samples
Normal	94.8%
Function code recognition attack	0.19%
Response modification attack	0.1%
Force error in response attack	0.007%
Read attack	4.83%
Write attack	0.06%
Replay attack	0.006%

**Table 3:** Performance comparisons of the proposed method and the JC-AC method for the multi-class case.

Method	Precision	Recall	F1-Score
JC-AC [1]	0.9985	0.9978	0.9980
Proposed Method	0.9998	0.9999	0.9998

**Table 4:** Performance comparisons of the proposed method and related ICS anomaly detection methods for the binary-class (i.e., normal-anomalous) case.

Method	Precision	Recall	F1-Score
JC-AD [1]	1.0000	1.0000	1.0000
SVM [4]	0.9756	1.0000	0.9876
OCSVM [4]	0.9862	0.9856	0.9859
RF [4]	0.9877	0.9871	0.9874
IF [4]	0.8739	1.0000	0.9327
DNN [4]	0.9692	1.0000	0.9843
GAN + DNN [5]	-	0.98	-
Proposed Method	0.9999	0.9999	0.9999

## Data Availability

Not applicable.
